# Familial and Genetic Influences on the Common Pediatric Primary Pain Disorders: A Twin Family Study

**DOI:** 10.3390/children8020089

**Published:** 2021-01-28

**Authors:** David Champion, Minh Bui, Aneeka Bott, Theresa Donnelly, Shuxiang Goh, Cindy Chapman, Daniel Lemberg, Tiina Jaaniste, John Hopper

**Affiliations:** 1 Department of Pain and Palliative Care, Sydney Children’s Hospital, Bright Alliance Building, High St, Randwick, NSW 2031, Australia; aneeka_bott@hotmail.com (A.B.); theresajdonnelly@gmail.com (T.D.); grandite@gmail.com (S.G.); cindy.a.chapman@gmail.com (C.C.); me@drlemberg.com (D.L.); Tiina.Jaaniste@health.nsw.gov.au (T.J.); 2 School of Medicine, University of New South Wales, Kensington, NSW 2052, Australia; 3 Centre for Epidemiology and Biostatistics, University of Melbourne, Melbourne, VIC 3010, Australia; mbui@unimelb.edu.au (M.B.); j.hopper@unimelb.edu.au (J.H.); 4 Paediatric Gastroenterology, Sydney Children’s Hospital, High St, Randwick, NSW 2031, Australia

**Keywords:** primary pain, pediatrics, twin family study, familial, genetic, questionnaire survey

## Abstract

The primary pain disorders of childhood are highly prevalent but have infrequently been studied collectively. Genetic influences have been suggested to be causally implicated. Surveys were sent to 3909 Australian twin families, assessing the lifetime prevalence of growing pains, migraine, headache, recurrent abdominal pain, low back pain, and persistent pain (not otherwise specified) in pediatric twins and their immediate family members. Comparisons between monozygous (MZ) and dizygous (DZ) twin pair correlations, concordances and odds ratios were performed to assess the contribution of additive genetic influences. Random-effects logistic regression modelling was used to evaluate relationships between twin individuals and their co-twins, mothers, fathers and oldest siblings with the subject conditions. Twin analyses of responses from 1016 families revealed significant influence of additive genetic effects on the presence of growing pains, migraine, and recurrent abdominal pain. The analyses for headache, low back pain, and persistent pain overall did not conclusively demonstrate that genetic influences were implicated more than shared environmental factors. Regression analyses demonstrated varying levels of significance in relationships between family members and twin individuals for the tested conditions, with strongest support for genetic influences in growing pains and migraine. These data, together with previously published association analyses, suggest common causal influences including genes.

## 1. Introduction

The common recurrent and chronic pain disorders of childhood and adolescence without injury or definable disease, increasingly categorized as primary pain disorders, share several common features. These include spontaneous or easily provoked onset, female preponderance, no biomarkers or pathology identifiable by routine clinical or imaging investigations, disordered somatosensory processing in the central nervous system, multiple comorbidities or associations with each other and with additional disorders including anxiety and depression. They also tend to have multiple risk markers (associations which might be causal), risk of later-life adverse pain and psychological outcomes, and wide familial and community prevalence [1,2,3,4]. Our broad objective in this twin family research program was to study potentially shared causal mechanisms between the common pediatric primary pain disorders through associations [4] and familial and genetic analyses.

The primary pain disorders of interest in this study are growing pains, migraine, non-migraine headache, recurrent abdominal pain and low back pain. Not all cases in the latter three categories are primary, but the questionnaires used in the current study were designed generally to exclude secondary conditions. A separate category, persistent pain not otherwise specified (NOS), mostly chronic pain disorders, has been included. The primary pain disorders have, in the past, mainly been studied individually. In this project, the pain disorders have been studied collectively, enabling insight into shared comorbidities [4] and comparative genetic influence.

A prerequisite for shared causal mechanisms in the common primary pain disorders is the demonstration of associations or comorbidities between them. The analyses from the previously published component of the twin family study [4] found independent associations between growing pains, migraine, non-migraine headaches, and recurrent abdominal pain; also independent associations between those primary pain disorders (except for growing pains) and persistent pain disorders; and independent associations between growing pains and persistent pain and restless legs syndrome; and between migraine and persistent pain with iron deficiency. Low back pain was not included in that publication. The extent and strength of the associations supported our hypothesis of causal mechanisms in common.

An important further prerequisite to the exploration of common causal influences is familial prevalence within and across the primary pain disorders, and genetic influence hypothesized from twin and genomic studies. There has been an intense recent interest in parental transmission of pain, commonly chronic pain [5,6,7,8,9,10,11,12,13,14,15]. These studies confirm familial relationships for pain and focused on biopsychosocial factors across the generations, with barely a mention of the potential for genetic transmission. Likewise, a systematic review with meta-analysis of musculoskeletal pain [16] provided no content about genetic risk.

The principal aim of this study was to investigate, using a twin family design [17,18], the hypothesis that the common primary pain disorders of childhood are substantially familial and potentially genetically influenced. The widely used twin pair studies include both monozygotic and dizygotic pairs, and invoke the assumptions of the classic twin model, the null hypothesis that genetic factors do not have a role in explaining variation in a trait that can be tested. By studying the relatives (mother, father and eldest sibling) of twins, important extra information on familial traits can be obtained.

Twin and genomic studies have provided evidence for genetic factors in non-specific or musculoskeletal multiregional and widespread pain disorders [19,20], migraine [21,22,23], functional (primary) gastrointestinal disorders especially irritable bowel syndrome [24], and low back pain [25]. Results for non-migraine headache have been equivocal. Phenotypic heterogeneity has been a problem in the studies, especially for tension-type and unclassified headaches, and for the functional or primary gastrointestinal disorders. Evidence for a significant genetic role in pediatric pain studies has, overall, been sparse and less conclusive.

## 2. Materials and Methods

### 2.1. Recruitment

Respondents were recruited via the Twins Research Australia (TRA) database. TRA is a national volunteer resource of twin pairs and higher-order multiples willing to consider participating in health, medical, and scientific research [26]. TRA reports that response rates are typically between 4 and 68% depending on study criteria and involvement requirements. Further information about the TRA can be found at https://www.twins.org.au/research/twin-and-data-resource/70-membership.

Random selection was determined by randomly sorting/extracting ID numbers unrelated to the date of TRA registration or any other details. Random selections were made for each zygosity/sex group separately aiming for an even number of MZ/DZ pairs and males/females pairs.

### 2.2. Participants and Procedure

Families with twins aged 3 to 18 years, who had not been approached to participate in other TRA studies in the previous 6 months, were mailed invitations to take part in the current study. Accepting families were mailed questionnaire assessments for growing pains, migraine, non-migraine headache, recurrent abdominal pain, low back pain and persistent pain (NOS) disorders. Low back pain questionnaires were sent in a second phase. The wide pediatric age range selected for this study was determined by considering the differing peak ages of incidence and prevalence of the primary pain disorders. Growing pains age range is 3–12 years [27], peaking at 4–6 years when it forms a common triad with headaches and recurrent abdominal pain [28], while migraine [29] and low back pain [30] are more prevalent in later adolescence. This was a pediatric study, hence the upper age limit of 18 years.

In each questionnaire pack, separate forms were provided for completion by mothers, fathers, twin individuals and the oldest non-twin sibling. Parents responded to questions about previous diagnoses, notably including history of iron deficiency, and answered questions for 3–5-year-old children. For all questions covering 6–18-year-old children, the responses were determined by a parent and child together.

Zygosity was recorded via reference to available results of DNA testing or via a 10-item questionnaire assessing similarities between twin pairs [31].

Ethics approval for this study was obtained from the Human Research Ethics Committee at the South Eastern Sydney and Illawarra Area Health Service of New South Wales, Australia.

### 2.3. Diagnostic Categorization of the Pain Disorders

Growing pains. Diagnostic criteria for growing pains were derived from Evans [27], Peterson [32], and previously applied by Champion et al. [33]. Individuals classified as meeting criteria for growing pains were those that endorsed four essential criteria (bilateral nature of pain, onset between 3 and 12 years of age, pain typically occurring at night, and no significant limitation of activity or limping) and denied the presence of exclusion criteria (evidence of orthopedic disorder, abnormalities in any specific tests performed, e.g., x-rays, bone scans).

Migraine. Diagnostic criteria for migraine with and without aura, as defined by the International Headache Society Classification Subcommittee [34], modified for pediatric application [35]. Participants were classified as having a migraine condition if they endorsed all criteria specified on the scale (at least 5 attacks fulfilling criteria, lasting 4–72 h, at least 2 of the following: unilateral location, pulsating, moderate/severe pain intensity, aggravation by or causing avoidance of routine physical activity, and during headache, nausea/vomiting, and/or photophobia and phonophobia). There was no separate classification made for individuals who indicated experiencing visual or other aura.

Headache. Participants were classified as having a headache condition if they failed to meet the above criteria for migraine, however responded positively to the screening question ‘Have you/Has your child ever experienced recurring headaches?’ and indicated that episodes lasted 4–72 h—untreated or unsuccessfully treated.

Recurrent Abdominal Pain. In the same format as above, respondents were asked to indicate whether they/the subject child have had ‘recurrent abdominal pain (including irritable bowel)’. Subsequent questions relating to whether this was doctor diagnosed, age of onset and resolution were completed if responding positively. The Rome IV criteria for functional gastrointestinal disorders [36] was subsequently completed by questionnaire to families in which at least one twin had indicated a positive response to recurrent abdominal pain criteria for more definitive categorization. In a subset analysis of 92 responding families, the most common category was functional abdominal pain (23.9%), followed by abdominal migraine (22.9%) and functional dyspepsia (6.5%). The remaining 45.6% did not satisfactorily enable classification by Rome IV criteria.

Low Back Pain. Respondents were classified as having low back pain if they responded positively to the question ‘During your lifetime “Have you” or “Has your child ever had pain in their low back in the shaded area, which lasted for at least 3 months?’ Participants received a body map with shading in the area between the lower ribs and the lower gluteal folds to assist with recognizing the subject area. If this question was answered positively, further information regarding the cause of the pain (injury, disease or without obvious reason), the age of onset, whether the pain spread down the leg, if the pain limited daily activities and the frequency of low back pain in the past 6 months (daily, weekly, monthly). Individuals were classified as cases for chronic low back pain if they experienced the condition for at least 3 months during their lifetime.

Persistent Pain (not otherwise specified). The screening questions, ‘Have you had’ or ‘Is it known that your child has (or has had) the following conditions’: ‘widespread pain/fibromyalgia’ or ‘other chronic or recurrent painful disorder (especially back or neck pain lasting more than 6 weeks)’ were responded to. If yes was indicated, participants were asked to specify. The responses were subsequently classified according to the proposed classification for International Classification of Diseases-11 (ICD-11) of the WHO [37]. Classification was completed by a student researcher together with a senior team member. Where this was not a duplication of another disorder assessed in the survey, participants were positively classified in the persistent pain category. Most cases had pain duration greater than 3 months.

### 2.4. Statistical Analyses

Summary statistics for DZ and MZ twins, as well as for siblings and parents, are presented by sample size and percentage, separately for cases and controls for each disorder. The controls refer to numbers without the specified pain condition (they are not pain-free controls). To compare the difference in cases between MZ and DZ twins, we used random-effects logistic regression [38] to estimate odds ratios, using DZ twins as reference category, adjusted for age and gender, and used Wald method for testing significant of odds ratios.

To determine whether a pain disorder was influenced by genetic and/or environmental factors, we used casewise concordance [39] analyses. Casewise concordance is the conditional probability of a twin being affected given that the co-twin is also affected. A greater concordance among MZ twins than DZ twins, under the assumption of classical twin model, which assumes MZ and DZ twins share same non-genetic factors, is evidence against a non-genetic influence hypothesis, in favour of a genetic influence. The maximum likelihood method was used to estimate the parameter and its standard error. The difference in parameters between MZ and DZ twins was then tested using the likelihood ratio statistics.

In addition to casewise concordance, for sensitivity analysis, we also used the correlations [40,41] and odds ratio (OR) methods [42] to compare similarity between MZ and DZ twins, unadjusted and adjusted for covariates, using the likelihood ratio test. The twin OR is the ratio of the odds of having a condition, given that the co-twin has the condition, to the odds of having the condition given that the co-twin does not have condition.

Random-effect logistic regression [38] was again used to investigate association between a twin and those of family members (sibling, mother and father) for each pain disorder. This was done by randomly selecting a twin from a twin pair where data must be available for all family members. The analyses were first conducted univariately, looking at association with each family member alone, but adjusted for age and gender whenever significant, and then multivariately by incorporating all family members into one model. The significant of association was tested using the Wald statistics.

Correlation and concordance were analyzed using our own programs written in R software package (http://www.R-project.org/) and are available if requested (mbui@unimelb.edu.au), while all other analyses were carried out using STATA software, version 13.1 (StataCorp LP, College Station, TX, USA).

## 3. Results

Questionnaires were sent to 3909 randomly selected twin families who met inclusion criteria. At the initial phase of survey distribution, twins’ ages ranged from 3 to 18 years (M (mean) = 10.46; SD (standard deviation) = 5.02), siblings’ ages from 4 to 38 (M = 12.63; SD = 17.16), mothers’ ages from 24 to 64 (M = 42.64; SD = 6.31) and fathers’ ages from 26 to 69 (M = 44.80; SD = 6.45). Low back pain was added 12 months later to the questionnaires sent to the same families. There were 1016 evaluable family responses received (overall response rate 26%).

Summary statistics. The twin data consisted of almost equal numbers of MZ and DZ twin pairs (MZ pairs = 504 (49.5%); DZ pairs = 513 (50.5%)), aged 3–18 years, and 51.2% were females. The MZ and DZ twins were well matched for age and gender (all *p* > 0.20). The number of twin individuals with each pain condition, stratified by zygosity, is given in Table 1. There were no differences between MZ and DZ twins for pain conditions (all *p* > 0.15). The number of cases and controls, and their percentage, for mother, father and oldest sibling are given in the Table 2.

Casewise concordance, correlation and odds ratio analysis. Casewise concordance, unadjusted for age and gender, showed consistently higher similarity in MZ than in DZ twins, suggesting that genetic factors influenced the variability for each of the pain conditions (Table 3) except headache (*p* = 0.064). Correlation and odds ratio methods produced results generally consistent with the casewise concordance (Table 4). However, persistent pain and low back pain results were not significant (*p* > 0.05) after adjustment for age and gender.

Univariate analysis association of twin individual with family member. Univariate analysis was first performed to find associations between each pain condition for a twin and co-twin, sibling, mother and father. Results for families with complete data are presented in Table 5. For all conditions, a twin was highly associated with his/her co-twin (all *p* < 0.001), and to a lesser extent with other family member, depending on the condition. A twin individual had statistically significant associations with the oldest sibling for growing pains, headache and persistent pain; significant associations with mother for all pain conditions; and significant associations with father for growing pains, recurrent abdominal pain, persistent pain and low back pain, while migraine association was marginal (*p* = 0.056).

Multivariate analysis. The analyses were then extended to the multivariate variables by incorporating all family members into one model, and results were also presented in Table 5. Mother and father have independent and additives effect on twins (all *p* ≤ 0.005) for migraine, while sibling and father were associated with twins in growing pains and persistent pain (all *p* ≤ 0.015), and only in mother for recurrent abdominal pain (*p* < 0.001). No additive association between twins and family members was observed for headache and low back pain.

## 4. Discussion

The principal aim of this study was to investigate, using a twin family design [18,19], the hypothesis that the common primary pain disorders of childhood are substantially familial and potentially genetically influenced. Recent publications on parental transmission of pain disorders [5,6,7,8,9,10,11,12,13,14,15], most commonly chronic pain, have focused on environmental influences, seeking factors which may be influenced to the benefit of the child (and parent). Parental transmission also includes genetic influences, and that has been the focus of this study. Each of the five most common primary pain conditions, also diverse persistent pain disorders, were shown to have familial associations (Table 5). The associations between the pain condition in twin individuals and the same condition in one or both parents were strongest for migraine, growing pains, recurrent abdominal pain and persistent pain. Associations with oldest siblings were generally weaker except for growing pains and persistent pain. Phenotypic diversity, including cases which were not primary pain, was strongest for persistent pain, low back pain and for recurrent abdominal pain, especially in the parents.

The strongest evidence for genetic influence (Table 3 and Table 4) was for migraine, growing pains and recurrent abdominal pain. For headache, low back pain and persistent pain disorders, the statistical significance in the correlation and odds ratio analyses became marginal or negative at the *p* < 0.05 level when controlling for age and gender.

The results of this twin family study will next be discussed regarding the individual primary pain disorders and then regarding the disorders collectively.

Growing pains is a familial disorder [43]. Under the assumptions of the classical twin study methodology [18], the results of the twin analyses by three different methods considered together with the family members’ analyses imply probable genetic influence on the variability of growing pains. This result strengthens the genetic inference of our previous twin family study involving a different sample [33]. Subsequent to the analysis presented in the current study, a substudy drawing on the same data has shown that one-third of the cases fulfilling the stated criteria for growing pains have the painful phenotype of the restless legs syndrome [44]. When urge to move the legs was used as an exclusion clause, a purer growing pains phenotype was identified which retained genetic influence.

Migraine concordance in the MZ twins was significantly greater than in DZ twins, and this evidence for probable genetic influence was shown also in the correlation and odds ratio analyses (adjusted for age and gender). Migraine in twin individuals was associated in multivariate analyses with migraine in both parents (again adjusted for age and gender). Migraine had the strongest evidence for genetic influence of the primary pain disorders in this study. While there have been numerous twin studies supporting genetic influence on migraine with or without aura [21,22,23,45,46,47,48,49,50,51,52,53,54,55], we have identified only one pediatric study, in 8- and 9-year-old children [56], a narrow age range. Our study provides additional twin family evidence for genetic influence in the pediatric population over a wide age range. The heritability estimate for migraine is in the range 0.34–0.57 [21]. Evidence overall, including genomic studies, for polygenetic factors in migraine is relatively strong, more so than for other common primary pain disorders. With respect to common polygenic migraine, genome-wide association studies have been reported to have identified 47 variant loci significantly associated with migraine risk [51,52]. We are unaware of any genomic studies in children and adolescents.

Primary non-migraine headaches (mostly tension-type) were shared by more MZ than DZ twins, but the difference did not reach statistical significance. Familial headaches were limited to the co-twin and only univariately to the eldest sibling and mother. Evidence of genetic effect in frequent tension-type headaches was found in a Danish twin study [57]. The same investigators found moderate genetic contribution to the variance in tension-type headaches in a large population sample of twins aged 12 to 41 years [58]. A genome-wide association study [59] found genetic associations with broadly-defined headache and that many psychological traits have genetic correlations with headache. There have been no pediatric twin or genomic studies specifically in non-migraine headache. There is evidence that people with migraine are more vulnerable to non-migraine headache [57], and because our sample of non-migraine headache specifically excluded those with migraine, it is probable that this has influenced our low estimate of genetic influence in non-migraine headache. A larger sample in our study might have shown statistically significant twin and family results, but any genetic effect on pediatric primary headache would have been assessed as minor.

Migraine and tension-type headaches are often viewed as distinct entities and were defined as such in the International Classification of Headache Disorders Edition 2. However, Ligthart et al. [50] showed that migraine and tension-type headache have partly shared etiologies (genetic and environmental). A rigid distinction is now probably not advisable, as indicated in the International Classification Edition 3 (2019): “The diagnostic difficulty most often encountered among the primary headache disorders is in discriminating between tension-type headache and mild forms of migraine without aura. This is more so because patients with frequent headaches often suffer from both disorders.”

Recurrent abdominal pain was shared by MZ twins significantly more frequently than by DZ twins by all three methods, implying probable genetic influence. Recurrent abdominal pain (lifetime prevalence) was significantly shared by mothers in the multivariate analyses which controlled for sex/gender and age, but only shared with fathers in the univariate analyses. Recurrent abdominal pain is a descriptive term and not a diagnosis, and has been defined as at least three episodes of pain occurring over at least three months that are severe enough to affect daily activities [60]. Recurrent abdominal pain in children is otherwise termed functional abdominal pain [61], psychogenic or nonspecific abdominal pain, and in ICD-11 pain terminology when chronic, primary abdominal (or visceral) pain. In Rome IV criteria, the subclassifications are abdominal migraine, functional abdominal pain, functional dyspepsia and irritable bowel syndrome. In our subset analysis of the children with recurrent abdominal pain using Rome IV criteria [36] the categories were mainly functional abdominal pain, abdominal migraine, unclassified, and a small percentage with functional dyspepsia. No doubt the parents with recurrent abdominal pain were diagnostically heterogeneous, and genetic transmission could not be validly claimed. There is evidence, albeit limited, for genetic factors in pediatric RAP. A family study on functional gastrointestinal disorders [62] implies a genetic influence on pediatric RAP, and a population-based study [63] showed that genetic variation in the *NPSR1* gene, which codes for the neuropeptide S receptor, influences children’s predisposition to RAP. There have been no twin studies published on pediatric RAP, and the present study further suggests a potential role for genetic influence.

The heritability of low back pain assessed from the twin data was marginal, with implications of probable genetic influence from the casewise concordance and univariate odds ratio and correlation data, but the latter two MZ/DZ differences did not quite hold in the analyses controlling for age and gender, which could be due to a type 2 error. Parental low back pain history in association with the twin individuals was positive only for the univariate analyses. There is some evidence for genetic influence on non-specific low back pain in adult twin studies [64,65,66,67], adult genome-wide meta-analyses [26] and from mixed samples which included adolescents [68]. Evidence for genetic influence on non-specific low back pain in children and adolescents is less conclusive. A population-based epidemiological study of schoolchildren aged between 13 and 15 years did show increased risk if there was a positive family history [69]. The one published pediatric population-based twin study in low back pain [70], in 11-year-old Finnish children, found that genetic factors played at most a minor role. A heritability analysis among twin pairs aged 12–22 years revealed that the life-time prevalence of low back pain had a genetic component [71]. It might be that genetic risk for low back pain increases across adolescence into early adulthood.

Our heritability assessment for persistent pediatric pain disorders drawn from our casewise concordance and univariate odds ratio and correlation data was marginal, in keeping with conflicting evidence in the literature. Parental chronic pain was associated with chronic non-specific pain and chronic multisite pain in adolescents and young adults (13–18 years) in a cross-sectional population survey in Norway [72]. A genome-wide study of multisite chronic pain in the UK [73] showed results consistent with chronic multisite pain having a significant polygenic component, with a single-nucleotide polymorphism (SNP) heritability of 10.2%. In contrast, other studies have shown no parent–child associations [74,75].

The painful phenotype of restless legs syndrome and primary dysmenorrhea qualify as primary pain disorders. We have shown, in prior publications, that they are also familial, with a probable genetic component [76,77].

Collectively, the primary pediatric pain disorder life prevalence for each condition as determined in this study was well within the published population prevalence range. The most commonly determined primary pain disorders in the children and adolescents (growing pains, headache and recurrent abdominal pain) is in accord with published prevalence data [28]. The prevalence rates of the pain disorders in the parents were also as expected. The twin pair analyses showed consistently higher degrees of similarity in MZ than in DZ twins for growing pains, migraine and recurrent abdominal pain (and also in painful restless legs syndrome and primary dysmenorrhea in derivative studies cited above) consistent with genetic factors influencing the variability. The twin pair analyses for heritability of headaches and low back pain were marginal, with loss of statistical significance when controlling for age and gender, consistent with other publications and we conclude that evidence for genetic influence was inconclusive.

Considering the genetic influence results in this study together with the associations between the primary pain disorders [4], we suggest the following implications:

- Shared causal mechanisms, including shared genes, may contribute to the common primary pediatric pain disorders [78]. A genome-wide association study could be used to investigate this hypothesis.
- In addition to comprehensive enquiry into familial environmental influences, it is potentially beneficial in the management of a pediatric patient with recurrent or chronic pain for clinicians to review thoroughly the individual and family history for other pain disorders and for restless legs syndrome and iron deficiency [4].

Twin studies are valuable in achieving initial evidence for genetic influence [18,79], though they do have their limitations. Our cross-sectional design limits causal conclusions. The diagnoses in our study were also based on parent and self-report questionnaires, rather than face-to-face medical interviews, this being inevitable in a large-scale epidemiological survey. The diagnostic categorization was thus likely rather than confirmed, lacking some of the checks and balances of a consultation by a specialized clinician. The category of persistent pain may be considered too heterogeneous for meaningful analysis, especially given the sample size. The response rate was relatively low at 26%. We were unable to control for recall bias. We have not explored environmental factors or gene–environment interactions. Finally, the sample size was insufficient to avoid the risk of a type 2 error in the estimation of familial and genetic factors in non-migraine headache, low back pain and persistent pain, but any genetic contributions have probably been minor compared to environmental influences. Therefore, we have taken a conservative view and have not submitted heritability estimates. There was a requirement for wide age ranges, as explained in the Methods.

The strengths of this study included the multiplicity of the primary pediatric pain disorders (unprecedented in a twin study), the twin family methodology, and the extent of the information from a questionnaire survey (although this limited the numbers of responding families).

von Baeyer and Champion [80] reviewed genetic and environmental factors which influence the vulnerability to pediatric pain and created a model which was influential in this twin family research [Supplementary Figure S1]. In the current study, the focus has been on potential genetic transmission of the common primary pain disorders of childhood collectively, so far as can be inferred by results of twin family analyses, while [5,6,7,8,9,10,11,12,13,14,15,16] were studies which focused largely on environmental psychosocial factors in chronic pain. Ideally, comprehensive genetic and environmental determinants of parental transmission of the common pediatric primary and chronic pain disorders could be researched by a large prospective twin family research design. However, our experience suggests that such a project would be exhausting for participants and compliance would be low. Realistically, future research needs to be relatively focused and clinical knowledge acquired progressively. The twin family design has substantial advantages [17].

## 5. Conclusions

This study has provided evidence in support of hypotheses of genetic influences in common pediatric primary pain disorders, at least in growing pains, migraine, and recurrent abdominal pain. When considered with our previous evidence for associations between these disorders and further evidence for shared genetic pathophysiological mechanisms among painful conditions, we hypothesize that causal mechanisms common to the primary pediatric pain disorders might include shared genes.

## Figures and Tables

**Table 1:** Summary statistics for DZ and MZ twins, and comparison of the difference between these two groups.

		MZ			DZ				
	Yes	No	%	Yes	No	%	OR	95% CI	*p*
GP	196	811	19.5	178	848	17.4	1.20	(0.75, 1.92)	0.454
Migraine	70	937	6.95	69	957	6.73	0.96	(0.55, 1.67)	0.881
Headache	141	866	14.0	133	893	13.0	1.09	(0.72, 1.64)	0.679
RAP	140	866	13.9	123	902	12.0	1.21	(0.80, 1.83)	0.366
LBP	79	470	14.4	80	677	10.6	1.51	(0.85, 2.66)	0.158
PP	68	938	6.76	59	966	5.76	1.14	(0.66, 1.98)	0.637

GP = growing pains; RAP = recurrent abdominal pain; LBP = low back pain; PP = persistent pain not otherwise specified (NOS), mostly chronic; No = number without the specified condition (not pain-free controls); % = percentage with condition; OR = odds ratios compare DZ twins (preference group) to MZ twins, adjusted for age and gender whenever significant; *p* = *p*-value using the Wald test.

**Table 2:** Sample size and percentage with each pain condition for mother, father and oldest sibling, separately for cases and controls.

	Mother		Father		Sibling	
	Case (%)	Control (%)	Case (%)	Control (%)	Case (%)	Control (%)
GP	238 (23.5%)	775 (76.5%)	116 (12.6%)	806 (87.4%)	123 (19.5%)	508 (80.6%)
Migraine	165 (16.3%)	848 (83.7%)	42 (4.6%)	879 (95.4%)	18 (2.9%)	608 (97.1%)
Headache	369 (36.4%)	644 (63.6%)	170 (18.5%)	751 (81.5%)	121 (19.3%)	505 (80.7%)
RAP	231 (22.8%)	782 (77.2%)	99 (10.8%)	814 (89.2%)	41 (6.6%)	585 (93.4%)
LBP	273 (42.4%)	371 (57.6%)	229 (40.5%)	336 (59.5%)	50 (12.2%)	361 (87.8%)
PP	192 (19.0%)	819 (81.0%)	117 (12.8%)	795 (87.2%)	44 (7.0%)	588 (93.0%)

GP = growing pains; RAP = recurrent abdominal pain; LBP = low back pain; PP = persistent pain (NOS); No = number without the specified condition (not pain-free controls); Control = number without the specified condition (not pain-free control).

**Table 3:** Casewise concordance (C) analysis for MZ and DZ twins for each pain condition.

	MZ					DZ					
	*N_c_*	*N_d_*	*N*_00_	C*_MZ_*	95% CI	*N_c_*	*N_d_*	*N*_00_	C*_DZ_*	95% CI	*p*
GP	61	74	368	0.62	(0.54, 0.70)	41	96	376	0.46	(0.37, 0.55)	0.009
Migraine	16	38	449	0.46	(0.31, 0.60)	8	53	452	0.23	(0.10, 0.36)	0.024
Headache	33	75	395	0.47	(0.37, 0.57)	22	89	402	0.33	(0.23, 0.43)	0.064
RAP	33	73	396	0.48	(0.37, 0.58)	17	88	407	0.28	(0.17, 0.38)	0.009
LBP	19	41	213	0.48	(0.35, 0.62)	10	59	308	0.25	(0.13, 0.38)	0.016
PP	14	40	448	0.41	(0.26, 0.56)	6	47	459	0.20	(0.07, 0.34)	0.043

GP = growing pains; RAP = recurrent abdominal pain; LBP = low back pain; PP = persistent pain (NOS); *N_c_* *=* number of twin pairs concordant with condition; *N_d_* = number of twin pairs discordant for condition; *N*_00_= number of twin pairs where neither twin has condition; *p* = *p*-value comparing the difference between MZ and DZ twins casewise concordance using the likelihood ratio test.

**Table 4:** Correlation (*Corr*) and odds ratio (OR) analysis for MZ and DZ twins for each condition.

	Correlation Analysis						OR Analysis					
	MZ		DZ				MZ		DZ			
	*Corr_MZ_*	95% CI	*Corr_DZ_*	95% CI	*p* *	*p* **	OR*_MZ_*	95% CI	OR*_DZ_*	95% CI	*p* *	*p* **
GP	0.53	(0.43, 0.62)	0.35	(0.25, 0.45)	0.009	0.013	16.4	(9.6, 28.0)	6.42	(3.8, 10.8)	0.017	0.013
Migraine	0.42	(0.27, 0.55)	0.18	(0.06, 0.32)	0.022	0.025	16.4	(7.0, 38.2)	3.89	(1.5, 10.0)	0.027	0.025
Headache	0.38	(0.27, 0.49)	0.23	(0.13, 0.34)	0.062	0.056	7.53	(4.1, 13.8)	3.16	(1.7, 5.96)	0.085	0.051
RAP	0.39	(0.28, 0.50)	0.18	(0.08, 0.30)	0.011	0.013	9.77	(5.4, 17.6)	3.4	(1.8, 6.4)	0.022	0.016
LBP	0.39	(0.25, 0.53)	0.17	(0.05, 0.31)	0.030	0.138	7.24	(3.2, 16.2)	3.11	(1.36, 7.1)	0.016	0.150
PP	0.37	(0.23, 0.51)	0.15	(0.05, 0.31)	0.047	0.076	14.4	(6.1, 33.8)	4.74	(1.7, 12.9)	0.043	0.098

GP = growing pains; RAP = recurrent abdominal pain; LBP = low back pain; PP = persistent pain (NOS); CI = confidence interval; *p* = *p*-value * unadjusted and ** adjusted for age and gender using likelihood ratio test.

**Table 5:** Associations between twin individual’s condition with family member’s condition (adjusted for age and gender whenever significant).

			Co-Twin		Sibling		Mother		Father	
Twin	Analysis	N	OR	*p*	OR	*p*	OR	*p*-Value	OR	*p*
GP	Univ	631	12.9	<0.001	4.65	<0.001	3.19	<0.001	2.67	<0.001
	Mult	631	9.29	<0.001	2.19	0.005	1.65	0.065	1.99	0.015
Migraine	Univ	626	10.2	<0.001	2.92	0.101	4.52	<0.001	2.81	0.056
	Mult	626	7.60	<0.001	1.51	0.702	3.01	0.005	1.66	<0.001
Headache	Univ	626	6.10	<0.001	2.64	<0.001	2.01	0.004	1.56	0.120
	Mult	626	5.22	<0.001	1.44	0.234	1.62	0.073	1.19	0.609
RAP	Univ	626	9.62	<0.001	2.06	0.076	2.51	<0.001	2.73	0.001
	Mult	626	8.13	<0.001	0.98	0.957	3.50	<0.001	1.83	0.081
PP	Univ	632	10.8	<0.001	6.88	<0.001	2.22	0.032	3.68	0.001
	Mult	632	8.62	<0.001	5.29	<0.001	4.56	0.272	3.00	0.011
LBP	Univ	283	6.84	<0.001	2.02	0.126	2.67	0.006	2.05	0.042
	Mult	283	5.38	<0.001	1.19	0.737	1.90	0.097	1.60	0.261

GP = growing pains; RAP = recurrent abdominal pain; LBP = low back pain; PP = persistent pain (NOS); Univ = univariate analysis; Mult = multivariate analysis; N = sample size; OR = odds ratio; *p* = *p*-value using the Wald test.

## Data Availability

The data presented in this study are available on request from the corresponding author.

## Supplementary Materials

The following are available online at https://www.mdpi.com/2227-9067/8/2/89/s1, Figure S1: Hypothesized antecedents and consequences of pain vulnerability [80].
