# Supplementary material for: Familial and Genetic Influences on the Common Pediatric Primary Pain Disorders: A Twin Family Study
Source: Children (Basel). 2021 Jan 28;8(2):89. doi: 10.3390/children8020089 (PMC7911833; doi:10.3390/children8020089)
Supplement: Supplementary file 1 [file children-08-00089-s001.pdf]

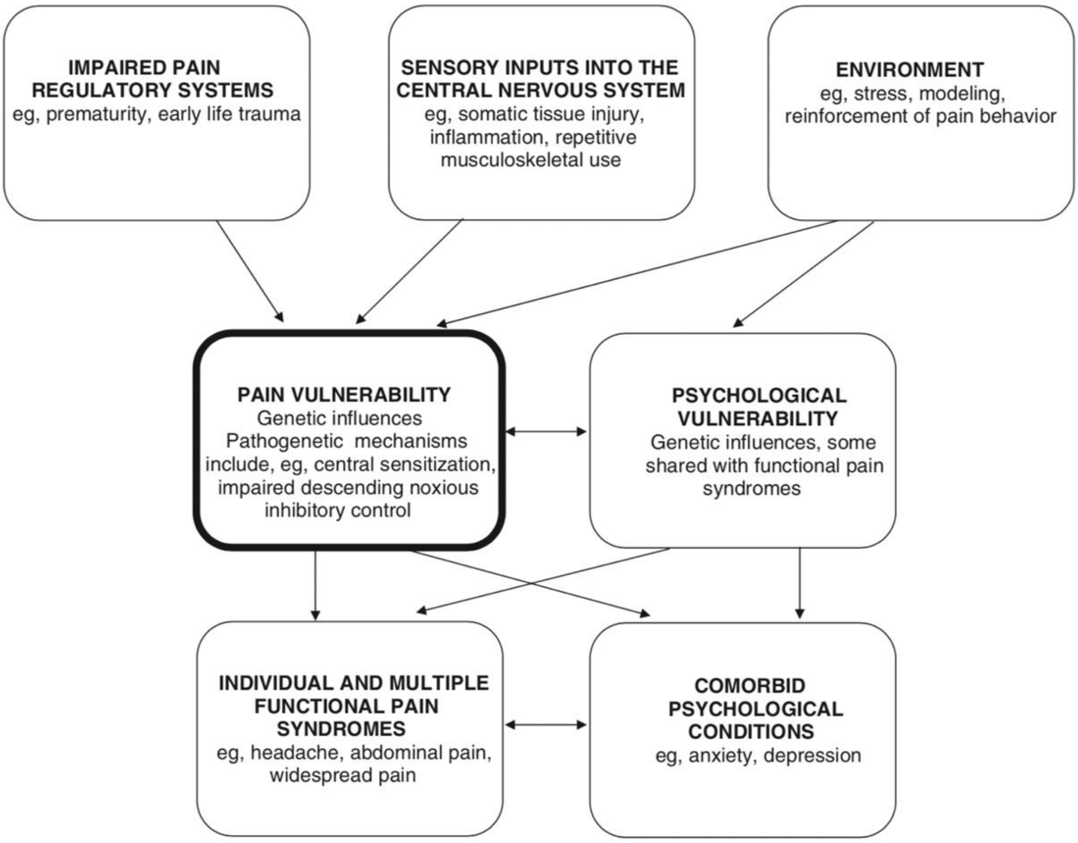

Figure S1. Key constructs discussed in the commentary, showing hypothesized antecedents and consequences of pain vulnerability. Reprinted with permission from ref. [80]. Copyright 2011 Oxford University Press.
